# Impact of Birth Companionship on Maternal Outcomes: A Retrospective Cohort Study at King Faisal General Hospital, Al-Ahsa, Saudi Arabia (2024)

**DOI:** 10.7759/cureus.100418

**Published:** 2025-12-30

**Authors:** Fatmah Meteb Alnofei, Rahma Baqer ALGadeeb, Hajar Alsalem, Ayat Abdulhadi Alabdullah, Shurooq Ahmed Alagnam

**Affiliations:** 1 Preventive Medicine Department, Al-Ahsa Health Cluster, Ministry of Health, Al Hofuf, SAU; 2 Maternity Research Unit, Maternity and Children Hospital, Ministry of Health, Al Mubarraz, SAU; 3 Obstetrics and Gynecology Department, King Faisal General Hospital, Ministry of Health, Al Hofuf, SAU

**Keywords:** anxiety, delivery, health facilities, natural childbirth, obstetric

## Abstract

Background

The World Health Organization emphasized the importance of having a birth companion for every woman during childbirth. However, the routine practice of allowing or providing a birth companion is still defective in several countries.

Objectives

This study aimed to assess the impact of childbirth companionship on maternal outcomes.

Methods

This retrospective cohort study included women giving birth at King Faisal General Hospital, Al-Ahsa, Saudi Arabia. The minimum sample size was calculated using G*Power for a two-tailed comparison of two independent means (α=0.05, power=80%, Cohen’s d=0.5), yielding a required sample of 130 women. To enhance statistical power and account for potential missing data, all eligible participants were included, resulting in a final sample of approximately 167 women. Women were categorized into an exposed group (experienced labor and delivery with a birth companion) and an unexposed group (without birth companionship). Primary outcomes included mode of delivery, duration of labor, time of initiating breastfeeding, and neonatal birth weight. Secondary outcomes included anxiety during labor. Statistical analyses were performed using R, with group comparisons conducted using appropriate parametric or nonparametric tests, followed by multivariable regression adjusted for key confounders (e.g., mother’s age, parity, and comorbidities) and false discovery rate correction for multiple testing. A p-value < 0.05 was selected as the significance level for the interpretation of statistical tests.

Results

This study included 167 women, out of whom 67 had a birth companion (40.1%). Women with a companion were younger, more educated, and more often primiparous. Birth companionship was not associated with labor duration, mode of delivery, neonatal outcomes, skin-to-skin contact, or breastfeeding initiation, but was associated with a higher rate of episiotomy (risk difference: 46%, 95% CI: 32%-61%, p<0.001). Women with a birth companion reported significantly lower overall anxiety scores compared to those without a companion (mean difference: -4.8, 95% CI: -6.4 to -3.2, p<0.001). After adjustment, having a birth companion was associated with a higher maternal anxiety-related total score (adjusted β=5.16, 95% CI: 3.32-7.00) and increased odds of episiotomy (adjusted OR=5.40, 95% CI: 2.06-14.87, p=0.003), while no significant associations were observed with prolonged labor, cesarean delivery, neonatal medical care, skin-to-skin contact, or delayed breastfeeding after correction for multiple testing.

Conclusion

The presence of a birth companion is associated with positive feelings of calmness and emotional support, as well as less anxiety during delivery. These findings support including birth companions in maternity care policies, with guidance to ensure safe and effective support, alongside routine clinical care.

## Introduction

The quality of care during childbirth represents a great concern worldwide owing to the potential consequences on maternal and neonatal health [1]. Continuous support and care are required to alleviate the fear and anxiety that women often experience during childbirth. The World Health Organization (WHO) has issued several guidelines, which recommend allowing and supporting the presence of a woman’s companion of her choice during childbirth to provide emotional and practical support [2,3]. A birth companion can be defined as “any person, chosen by a woman, to provide her with continuous support during labour and childbirth” [4]. This companion may be the woman’s partner, another family member, a trained supporter, or a nurse/midwife [5]. The role of a birth companion usually starts from early labor until at least the child's delivery takes place [6].

The roles of a birth companion can take various forms. Birth companions can contribute to the quality of care by facilitating the communication between mothers, families, and healthcare workers, which may positively impact the interactions between the mothers and the healthcare staff [7]. In addition, birth companions can enhance pain relief by applying massage, assisting mobility and position changes, providing emotional support and reassurance [5], and reducing anxiety [8].

Research has demonstrated that the presence of birth companions can ensure safe delivery and better maternal and neonatal outcomes. Reports have shown that women with no labor companions were more liable to experience feelings of helplessness and poor communication [9]. In addition, companionship during labor enhances spontaneous vaginal birth, shorter labor duration, better five-minute Apgar score, and higher satisfaction rates [10-12]. Moreover, research shows that childbirth companionship is linked to expedited postpartum recovery and enhances the bond between mothers and infants [11,13,14].

While the presence of a labor companion has become the standard care in several regions worldwide, many health systems have no official policies regarding this recommended practice [15]. Reforms in the Saudi healthcare system have been undertaken within the maternity care services to reduce the high health expenditure arising from the increased rate of unnecessary medical/obstetric interventions. The Saudi Ministry of Health (MOH) founded the Mother-and-Baby-Friendly Hospital Initiative in January 2018 to provide evidence-based, mother-and-baby-friendly maternity care and reduce the rate of interventions during childbirth. The MOH has issued several guidebooks from this perspective, including the “Companion’s Policy in the Obstetrics and Gynecology Departments” book [16]. However, most hospitals in Saudi Arabia do not allow the presence of birth companions, leaving women unattended due to staff shortages. These practices contrast with the WHO recommendations and the body of evidence that favors the continuous support by the woman’s selected companion. The few studies conducted in Saudi Arabia showed that only 14-43% of women report having ever had a birth companion, despite the fact that nearly half the women preferred birth companionship [17,18]. This gap between policy and practice in Saudi Arabia warrants further exploration to assess the benefits of birth companionship and the barriers that prevent its implementation.

A thorough evaluation of this initiative is critically needed to establish local evidence that supports or refines the existing national policies that recommend birth companionship as a standard element of respectful maternity care. The present study was conducted at King Faisal General Hospital (KFGH) in Al-Ahsa, Saudi Arabia, to provide context-specific evidence to inform policy and practice in maternity care in Saudi Arabia. Al-Ahsa is a prominent region located in the Eastern Province of Saudi Arabia and is one of the largest oases in the world [19]. With an estimated population of 1,067,691 [20], Al-Ahsa is known for its large and diverse community, making it a significant area for public health research. The study was carried out at KFGH, one of the main governmental referral hospitals in the region. The hospital was selected due to its high patient volume, particularly in obstetric cases, and its well-established maternity services. Its diverse patient population and comprehensive clinical records made it a suitable setting for evaluating maternal outcomes. Additionally, the accessibility of data and institutional support facilitated the implementation of this study.

The study aimed to examine the association of birth companionship with important maternal and neonatal outcomes. We hypothesized that birth companionship would be associated with more favorable maternal outcomes, including unassisted vaginal delivery, shorter duration of labor, lower levels of anxiety, and earlier initiation of breastfeeding compared with having no birth companion. In addition, we hypothesized that birth companionship may be associated with good neonatal outcomes in the form of normal birth weight and normal health status. Therefore, the specific study objectives were to (a) explore the barriers that hinder the implementation of birth companionship and (b) assess the association of having a birth companion with key maternal and neonatal outcomes. The study findings are expected to support evidence-informed decision-making, guide the scale-up of companionship practices across maternity care settings in Saudi Arabia, and enhance the alignment of national maternity care practices with global standards of respectful, woman-centered care in accordance with Vision 2030.

## Materials and methods

Study design and settings

This retrospective cohort study was conducted at KFGH in Al-Ahsa, Saudi Arabia. The study design was selected due to its suitability to assess the association of birth companionship with maternal and neonatal outcomes in a real-world clinical setting. As the Safe Childbirth Initiative had already been implemented at the study hospital, routinely collected clinical and administrative records enabled the identification of two naturally occurring cohorts: women who underwent childbirth with a companion and those who did not. This design allows the assessment of multiple outcomes following a single exposure, while reflecting the actual clinical practice without influencing care provision. In addition, it may be ethically questionable to randomly allocate birth companionship if a prospectively carried interventional study is considered.

Study population

The study population included pregnant women who gave birth at KFGH, Al-Ahsa, during the period from January to December 2024. Women were categorized into two groups based on their documented participation in the Safe Childbirth Initiative. The Safe Childbirth Initiative at KFGH in Al-Ahsa, Saudi Arabia, aims to promote respectful maternity care by allowing women to choose a companion during labor. The Safe Childbirth Initiative is a health promotion intervention for maternal and newborn health, which was established in 2023 in KFGH by the Health cluster in Al-Ahsa as a contribution to the 10-Step Baby-Friendly Hospital Initiative (BFHI) to provide a better experience of childbirth for pregnant women [16,21]. The main service provided through this initiative is to allow pregnant women to have a companion during labor. Every pregnant woman in the prenatal clinic has been invited to participate in the safe childbirth initiative during her last trimester, which includes assisting pregnant women to select in advance a childbirth companion. Additionally, both the pregnant woman and her companion attend lectures about breastfeeding and are educated about the BFHI hospital policy regarding breastfeeding. Participation in the initiative was voluntary and determined during routine antenatal care, whereby all pregnant women attending the prenatal clinic in their last trimester were informed about the initiative and invited to enroll.

The exposed group in the present study consisted of pregnant women who participated in the Safe Childbirth Initiative and experienced labor and delivery with a birth companion. The unexposed group included pregnant women who declined participation in the initiative and underwent childbirth without companionship. Both groups received the standard routine obstetric care, which included usual intrapartum clinical monitoring and obstetric management by healthcare staff.

Inclusion and exclusion criteria

The study included all women who delivered at KFGH between January 1 and December 31, 2024. Eligible participants were women with singleton, full-term pregnancies, defined as a gestational age of 37-40 weeks, who received antenatal care at KFGH and had complete delivery and postnatal records available in the hospital database.

**Figure 1:**
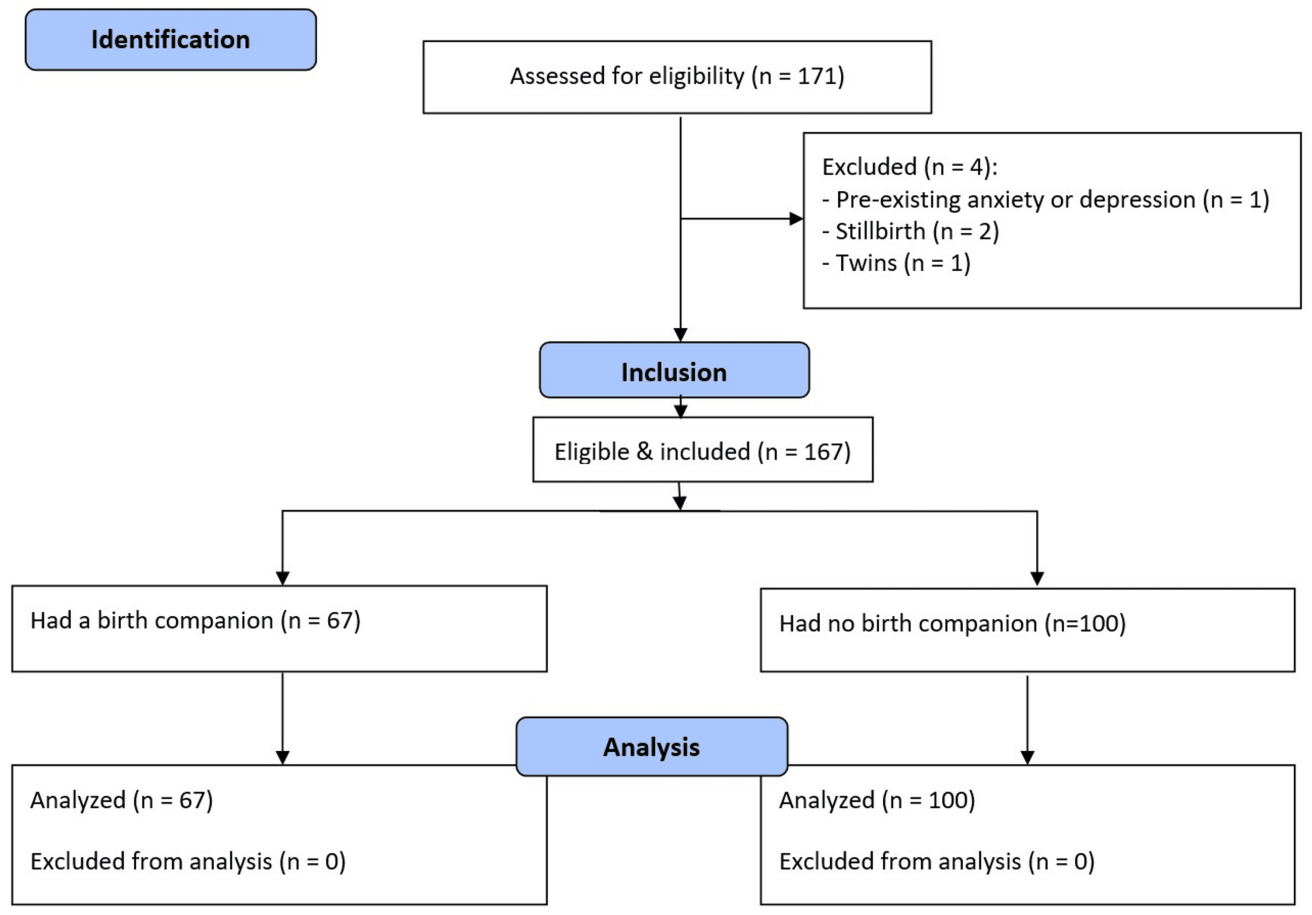
Participant selection flowchart

Women were excluded from the study if they had a documented pre-existing diagnosis of anxiety or depressive disorders before pregnancy, as these conditions could independently influence maternal and neonatal outcomes and confound the association between birth companionship and study outcomes. Women who experienced a stillbirth were excluded because maternal and neonatal outcomes in such cases differ substantially from those following live births and could bias outcome assessment. Participants who delivered outside KFGH were excluded to ensure consistency in clinical management, documentation practices, and accurate assessment of exposure to birth companionship. Additionally, women without documented postpartum follow-up were excluded due to the inability to reliably ascertain key maternal and neonatal outcomes, which could compromise data completeness and validity. Participants with incomplete data were excluded. A flowchart illustrating participant selection is provided in Figure 1.

Sample size

Based on the literature review, 43.2% of women in Al-Ahsa, Saudi Arabia, had a labor companion during delivery [18]. The minimum required sample size was calculated using G*Power software, employing the “Means: Difference between two independent means (two groups)” statistical test for the outcomes of anxiety scores and birth weight. The following parameters were used: a two-tailed test, an effect size (Cohen’s d) of 0.5, a group size ratio of 0.76 (43.2/56.8), an alpha level of 0.05, and a power of 0.8. The effect size of d=0.5 was selected based on the cutoff values for Cohen’s d, where values between 0.5 and 0.8 indicate a moderate effect size [22]. Based on these values, the minimum sample size was 74 for the non-companion group and 56 for the birth companion group (a total of 130 women).

However, to enhance the study's power and to account for potential missing or excluded data, the sample size was increased to include all available eligible participants (approximately 167 participants).

Data collection

Data Collection Tool

Data were collected using a structured data collection sheet developed based on a comprehensive literature review. The sheet included socio-demographic data (age, education level) and obstetrical characteristics (parity, gestational age at delivery).

Anxiety levels during labor were assessed using the Arabic version of the Modified State-Trait Anxiety Inventory (STAI), which was translated from the original English version and validated in prior studies [23,24]. To ensure cultural appropriateness and clarity, the Arabic version used in this study was reviewed by three specialists: an obstetrics consultant, a preventive medicine consultant, and a psychiatrist.

The STAI was used in this study to assess maternal anxiety during labor. It consists of 10 items rated on a 4-point Likert scale, with some items reverse-scored according to the instrument’s guidelines.

The data collection sheet was pilot-tested on a small sample of eligible participants (n=20) who were not included in the final analysis. The pilot testing aimed to assess the clarity, comprehension, and feasibility of the questions. Minor wording modifications were made based on participants' feedback to improve clarity.

Variable Definitions and Outcomes

Exposure variable: The primary exposure was birth companionship, defined as the presence of a chosen companion (e.g., spouse, family member, or other support person) with the woman during labor and/or delivery. Birth companionship status was recorded as a dichotomous variable (yes/no) based on maternal report during the phone interview.

Outcome measures: All outcomes were specified a priori based on clinical relevance and existing literature. Primary outcomes include maternal outcomes (mode of delivery, duration of labor), newborn outcomes (time of skin-to-skin, time of initiation of breastfeeding, and birthweight). All primary outcomes were extracted from hospital medical records. The secondary outcome included mother anxiety by the modified STAI, which was measured through maternal self-report during structured telephone interviews.

Covariates: Potential confounders were selected based on clinical relevance and prior literature and included maternal age, parity, and presence of comorbidities.

Data Collection Procedure

Data were collected through direct phone interviews with mothers, conducted by two trained research assistants. Before the start of data collection, the research assistants received standardized training on the study objectives, interview procedures, and ethical considerations to minimize interviewer bias. Responses were entered into a Google Form during the call. Data were then exported into Microsoft Excel (Microsoft® Corp., Redmond, WA) for coding and analysis. The timing of interviews after childbirth varied among the participants, ranging from about one month to six months postpartum. Exposure status (presence of birth companionship) was determined from hospital delivery records and was established before participant interviews

Data analysis

Statistical analysis was carried out using the R Statistical language (version 4.4.3; R Development Core Team, Vienna, Austria) [25]. Categorical variables (e.g., delivery mode, comorbidities) were summarized as counts and percentages. To enhance interpretability and avoid small cell counts, categories of selected categorical variables (e.g., labor duration, delivery mode, mother’s education) were regrouped into broader, clinically meaningful groups before analysis. Associations with the study groups were tested using Pearson’s chi-square test for independence of observations or Fisher’s exact test. Numerical variables (e.g., mother’s age, birthweight, anxiety scores) were assessed for normality using the Shapiro-Wilk test and visual inspection of Q-Q plots. Variables approximating a normal distribution were summarized as means with standard deviations and compared using unpaired t-tests, while variables showing deviation from normality were summarized using medians with interquartile ranges (IQR) and compared using the Wilcoxon signed-rank test. To account for potential confounding, multivariable regression analyses were performed, adjusting for clinically relevant covariates, including maternal age, parity, and presence of comorbidities. Results are reported as adjusted effect estimates with 95% confidence intervals. Given the presence of multiple primary maternal and neonatal outcomes, adjustment for multiple comparisons was applied using the Benjamini-Hochberg false discovery rate method. Complete-case analysis was employed; participants with missing outcome data were not included in the study.

Ethical considerations

The proposal was officially approved by the Institutional Review Board of Maternity and Children Hospital, Al-Ahsa Health Cluster (IRB MCH No. H-05-HS-137) with an IRB Log No. of 0102-EP-2025. All collected data were anonymous and confidential, to be utilized only for research. The study was conducted in accordance with the principles of the Helsinki Declaration.

## Results

The present study included 167 participants, out of whom 67 had a birth companion (40.1%) (Figure 1). The most frequent reported cause for not having a birth companion was that “the hospital did not mention it as a choice” (n=44, 44%), followed by “Having had repeated deliveries, I can handle it” (n=24, 24%). The age of the participants ranged between 19 and 47, with a mean of 30.4±6.1 years. Respondents without a birth companion had a significantly higher mean age than those with a companion (mean difference (MD): -4.5, 95% confidence interval (CI): -6.2 to -2.7, p<0.001). Most respondents (n=101, 60.5%) had a university degree, and 50 (29.9%) had a secondary school education only. Women with a birth companion showed a significant tendency to have higher education, particularly university or postgraduate degrees (risk difference (RD): 16%, 95% CI: 0.22%-32%, p=0.035). Most women were housewives (n=127, 76%). However, a significantly higher percentage of women who had a birth companion were still students compared to those without a companion (RD: 14%, 95% CI: 3.9%-24%, p<0.001). Approximately one quarter (n=42, 25.1%) of the participants were primiparous, while most participants were multiparous. There was a significant association between parity and having a birth companion, as a higher percentage of women with a birth companion were primiparous (RD: 45%, 95% CI: 31%-59%, p<0.001). Only 22 (13.2%) of the participants had a chronic medical disease. There were no significant associations between having a birth companion and having a chronic disease, previous abortion, a pregnancy-related complication/disease, or hospital admission (all p-values>0.05, Table 1).

**Table 1:** Participants’ sociodemographic characteristics and medical/obstetric history (total number=167) Abbreviation: CI = Confidence interval; MD: Mean difference; RD: Risk difference a n: Number; SD: Standard deviation; b: Welch two-sample t-test; c: Chi-squared test for trend in proportions; d: Fisher’s exact test; e: Pearson’s chi-squared test

Characteristic a	All participants N=167	Women with a childbirth companion, N=67	Women without a childbirth companion, N=100	Difference (95% CI) (MD/RD)	p-value
Sociodemographic characteristics					
Mother's age (Years), Mean ± SD (Range)	30.4 ± 6.1 (19.0 - 47.0)	27.7 ± 5.5 (19.0 - 47.0)	32.2 ± 5.8 (20.0 - 46.0)	-4.5 (-6.2 to -2.7)	<0.001*b
Mother's education, n (%)					
Primary/Intermediate school	16 (9.6%)	4 (6.0%)	12 (12.0%)	-6.0% (-16% to 3.7%)	0.035*c
Secondary school	50 (29.9%)	16 (23.9%)	34 (34.0%)	-10% (-25% to 4.9%)	
University graduate or postgraduate studies	101 (60.5%)	47 (70.1%)	54 (54.0%)	16% (0.22% to 32%)	
Mother's occupation, n (%)					
Housewife	127 (76.0%)	50 (74.6%)	77 (77.0%)	-2.4% (-17% to 12%)	<0.001*d
Student	11 (6.6%)	10 (14.9%)	1 (1.0%)	14% (3.9% to 24%)	
Employee	29 (17.4%)	7 (10.4%)	22 (22.0%)	-12% (-24% to 0.63%)	
Medical & obstetric history					
Parity, n (%)					
1st	42 (25.1%)	35 (52.2%)	7 (7.0%)	45% (31% to 59%)	<0.001*e
2nd or higher	125 (74.9%)	32 (47.8%)	93 (93.0%)	-45% (-59% to -31%)	
Previous abortion, n (%)	51 (30.5%)	15 (22.4%)	36 (36.0%)	-14% (-29% to 1.4%)	0.061e
Gestational diabetes, n (%)	12 (7.2%)	3 (4.5%)	9 (9.0%)	-4.5% (-13% to 4.2%)	0.365d
Anemia, n (%)	31 (18.6%)	15 (22.4%)	16 (16.0%)	6.4% (-7.2% to 20%)	0.298e
Hypertension, n (%)	9 (5.4%)	4 (6.0%)	5 (5.0%)	0.97% (-7.1% to 9.0%)	>0.999d
Preeclampsia, n (%)	8 (4.8%)	5 (7.5%)	3 (3.0%)	4.5% (-3.9% to 13%)	0.269d
Having a chronic disease, n (%)	22 (13.2%)	5 (7.5%)	17 (17.0%)	-9.5% (-20% to 1.4%)	0.074e
Hospital admission, n (%)	16 (9.6%)	5 (7.5%)	11 (11.0%)	-3.5% (-14% to 6.5%)	0.447e

The respondents had varying durations of labor. No significant association was observed between birth companionship and the duration of labor. Only 19 (11.4%) were delivered by a cesarean section. Episiotomy was done in 51 (30.5%), with a significantly higher percentage of participants with a companion compared to those with no companion (RD: 46%, 95% CI: 32%-61%, p<0.001), and the performance of episiotomy was significantly associated with primigravida (p<0.001, not shown in the tables). The weight of neonates ranged between 1.2 and 4.2 kg. The baby was male in 90 (53.9%) participants. Medical care was required for 56.9% (n=95) of neonates. Skin-to-skin contact was initiated in 47.3% (n=79) of participants. The initiation of breastfeeding was within one hour in 86 (51.5%) participants. No significant difference was found between participants with a companion and those without regarding the type of delivery, neonate’s weight, gestational age, sex, or need for care, the establishment of skin-to-skin contact, or delayed breastfeeding (all p-values>0.05, Table 2).

**Table 2:** Details of the delivery and mother and neonatal outcomes (total number=167) Abbreviation: CI = Confidence Interval a n: Number; SD: Standard deviation; b: Benjamini & Hochberg correction for multiple testing; c: *p<0.05; d: Pearson’s chi-squared test; e: Welch two-sample t-test; f: Fisher’s exact test # Hospital admission for neonatal monitoring and care, such as prematurity, respiratory support, or other routine medical observations

Characteristic a	All participants, N=167	Women with a childbirth companion, N=67	Women without a childbirth companion, N=100	Difference (95% CI)	p-value	q-value b,c
Duration of labor, n (%)						
≤12 hours	123 (73.7%)	46 (68.7%)	77 (77.0%)	-8.3% (-23% to 6.7%)	0.230d	0.368
>12 hours	44 (26.3%)	21 (31.3%)	23 (23.0%)	8.3% (-6.7% to 23%)		
Type of delivery, n (%)						
Vaginal	148 (88.6%)	63 (94.0%)	85 (85.0%)	9.0% (-1.2% to 19%)	0.072d	0.287
Cesarean	19 (11.4%)	4 (6.0%)	15 (15.0%)	-9.0% (-19% to 1.2%)		
Episiotomy, n (%)						
No	116 (69.5%)	28 (41.8%)	88 (88.0%)	-46% (-61% to -32%)	<0.001*d	<0.001*
Yes	51 (30.5%)	39 (58.2%)	12 (12.0%)	46% (32% to 61%)		
Neonatal outcomes						
Weight of baby (kg), Mean ± SD (Range)	3.0 ± 0.5 (1.2 - 4.2)	2.9 ± 0.5 (1.2 - 4.0)	3.0 ± 0.5 (2.0 - 4.2)	-0.10 (-0.25 to 0.06)	0.229e	0.368
Gestational age, n (%)						
32-37 weeks	5 (3.0%)	2 (3.0%)	3 (3.0%)	-0.01% (-5.3% to 5.3%)	>0.999f	>0.999
37-42 weeks	162 (97.0%)	65 (97.0%)	97 (97.0%)			
Gender of baby, n (%)						
Female	77 (46.1%)	28 (41.8%)	49 (49.0%)	-7.2% (-24% to 9.4%)	0.360d	0.48
Male	90 (53.9%)	39 (58.2%)	51 (51.0%)	7.2% (-9.4% to 24%)		
Need for neonatal medical care, n (%)						
No	72 (43.1%)	27 (40.3%)	45 (45.0%)	-4.7% (-21% to 12%)	0.548d	0.626
Yes	95 (56.9%)	40 (59.7%)	55 (55.0%)	4.7% (-12% to 21%)		
Skin-to-skin contact, n (%)						
No	88 (52.7%)	35 (52.2%)	53 (53.0%)	-0.76% (-17% to 15%)	0.923d	0.923
Yes	79 (47.3%)	32 (47.8%)	47 (47.0%)	0.76% (-15% to 17%)		
Delayed breastfeeding beyond 1 hour, n (%)						
No	81 (48.5%)	28 (41.8%)	53 (53.0%)	-11% (-28% to 5.4%)	0.155d	0.368
Yes	86 (51.5%)	39 (58.2%)	47 (47.0%)	11% (-5.4% to 28%)		

The companion in most cases was the mother or the sister of the participant (n=46, 68.7%). In 13 (19.4%) cases, the companion was the husband. The husband’s mother was mentioned by two (3%), while other relatives were companions in six (9%) cases. The most common form of support offered by the companion was emotional, through reassurance and communication with the medical staff (n=66, 98.5%). Companions also encouraged exercise and breathing in 24 (35.8%) cases. In 22 (32.8%) cases, the companion performed a massage for the mother.

The mothers’ feelings during delivery were assessed. The used tool had a good internal consistency, as demonstrated by a Cronbach’s alpha of 0.714 (95% confidence interval: 0.636-0.779), indicating reliable measurement of anxiety levels. As regards the positive feelings encountered during delivery, participants with birth companions showed lower scores for the items "I felt calm" (MD: -0.40, 95% CI: -0.72 to -0.08, p=0.011), "I felt emotionally supported" (MD: -0.90, 95% CI: -1.2 to -0.62, p<0.001), "I had less anxiety with a companion" (MD: -1.4, 95% CI: -1.7 to -1.1, p<0.001), and "I felt safe with a companion" (MD: -1.5, 95% CI: -1.7 to -1.2, p<0.001). The lower scores for these reverse-coded items indicated better emotional stability and less anxiety. Meanwhile, no significant differences were found between the two groups as regards the items assessing negative feelings (all p-values >0.05). Women with a birth companion had a significantly lower total score compared to those without a companion (MD: -4.8, 95% CI: -6.4 to -3.2, p<0.001), indicating lower anxiety levels (Table 3). The frequencies of the different answers for each item are presented in Tables 4-5.

**Table 3:** Comparison of the modified State-Trait Anxiety Inventory (STAI) anxiety score between the study groups (total number=167) Abbreviation: CI = Confidence interval; MD: Mean difference a IQR: Interquartile range; n: Number; SD: Standard deviation; b: Benjamini & Hochberg correction for multiple testing; c: *p<0.05; d: Wilcoxon rank sum test; e: Welch two-sample t-test

Characteristic a	All participants N=167	Women with a childbirth companion, N=67	Women without a childbirth companion, N=100	Difference MD (95% CI)	p-value	q-value b,c
Positive feelings						
I felt calm, median (IQR) (range)	2 (1 - 2) (1 - 4)	1 (1 - 2) (1 - 4)	2 (1 - 3) (1 - 4)	-0.40 (-0.72 to -0.08)	0.005*d	0.011*
I was able to control my emotions, median (IQR) (range)	2 (1 - 3) (1 - 4)	2 (1 - 3) (1 - 4)	2 (2 - 30 (1 - 4)	-0.25 (-0.58 to 0.09)	0.102d	0.16
I felt emotionally supported, median (IQR) (range)	1 (1 - 2) (1 - 4)	1 (1 - 2) (1 - 4)	2 (1 - 3) (1 - 4)	-0.90 (-1.2 to -0.62)	<0.001*d	<0.001*
I had less anxiety with a companion, median (IQR) (range)	2 (1 - 3) (1 - 4)	1 (1 - 1) (1 - 4)	3 (2 - 4) (1 - 4)	-1.4 (-1.7 to -1.1)	<0.001*d	<0.001*
I felt safe with a companion, median (IQR) (range)	2 (1 - 3) (1 - 4)	1 (1 - 1) (1 - 2)	3 (1 - 4) (1 - 4)	-1.5 (-1.7 to -1.2)	<0.001*d	<0.001*
Negative feelings						
I felt very stressed, median (IQR) (range)	3 (2 - 4) (1 - 4)	2 (1 - 3) (1 - 4)	3 (2 - 4) (1 - 4)	-0.32 (-0.66 to 0.03)	0.073d	0.134
I was afraid of the unknown, median (IQR) (range)	3 (1 - 4) (1 - 4)	3 (1 - 4) (1 - 4)	3 (1 - 4) (1 - 4)	0.08 (-0.29 to 0.46)	0.684d	0.753
I was worried about my baby's health, median (IQR) (range)	3 (2 - 4) (1 - 4)	3 (1 - 4) (1 - 4)	3 (2 - 4) (1 - 4)	-0.14 (-0.51 to 0.22)	0.444d	0.611
I was worried about making medical decisions, median (IQR) (range)	3 (1 - 4) (1 - 4)	2 (1 - 4) (1 - 4)	3 (1 - 4) (1 - 4)	-0.12 (-0.48 to 0.24)	0.517d	0.632
I was worried about my ability to tolerate the pain, median (IQR) (range)	3 (2 - 4) (1 - 4)	3 (2 - 4) (1 - 4)	3 (2 - 4) (1 - 4)	0.06 (-0.29 to 0.40)	0.794d	0.794
Total score						
Total score, Mean ± SD (Range)	23 ± 6 (10 - 37)	20 ± 5 (10 - 35)	25 ± 5 (14 - 37)	-4.8 (-6.4 to -3.2)	<0.001*e	<0.001*

**Table 4:** Positive feelings experienced by the studied women during labor (total number=167) a: Chi-squared test for trend in proportions

Characteristic	Overall, N=167	Women with a childbirth companion, N=67	Women without a childbirth companion, N=100	p-value
I felt calm, n (%)				
Not at all	23 (13.8%)	7 (10.4%)	16 (16.0%)	0.015*a
Somewhat	17 (10.2%)	5 (7.5%)	12 (12.0%)	
Moderately	54 (32.3%)	16 (23.9%)	38 (38.0%)	
Very much	73 (43.7%)	39 (58.2%)	34 (34.0%)	
I was able to control my emotions, n (%)				
Not at all	32 (19.2%)	11 (16.4%)	21 (21.0%)	0.149a
Somewhat	20 (12.0%)	8 (11.9%)	12 (12.0%)	
Moderately	63 (37.7%)	21 (31.3%)	42 (42.0%)	
Very much	52 (31.1%)	27 (40.3%)	25 (25.0%)	
I felt emotionally supported, n (%)				
Not at all	26 (15.6%)	2 (3.0%)	24 (24.0%)	<0.001*a
Somewhat	15 (9.0%)	1 (1.5%)	14 (14.0%)	
Moderately	37 (22.2%)	14 (20.9%)	23 (23.0%)	
Very much	89 (53.3%)	50 (74.6%)	39 (39.0%)	
I had less anxiety with(out) companion, n (%)				
Not at all	39 (23.4%)	2 (3.0%)	37 (37.0%)	<0.001*a
Somewhat	22 (13.2%)	3 (4.5%)	19 (19.0%)	
Moderately	28 (16.8%)	9 (13.4%)	19 (19.0%)	
Very much	78 (46.7%)	53 (79.1%)	25 (25.0%)	
I felt safe with(out) companion, n (%)				
Not at all	33 (19.8%)	0 (0.0%)	33 (33.0%)	<0.001*a
Somewhat	22 (13.2%)	0 (0.0%)	22 (22.0%)	
Moderately	30 (18.0%)	11 (16.4%)	19 (19.0%)	
Very much	82 (49.1%)	56 (83.6%)	26 (26.0%)	

**Table 5:** Negative feelings experienced by the studied women during labor a: Chi-squared test for trend in proportions

Characteristic	Overall, N=167	Women with a childbirth companion, N=67	Women without a childbirth companion, N=100	p-value
I felt very stressed, n (%)				
Not at all	40 (24.0%)	20 (29.9%)	20 (20.0%)	0.072a
Somewhat	41 (24.6%)	17 (25.4%)	24 (24.0%)	
Moderately	43 (25.7%)	17 (25.4%)	26 (26.0%)	
Very much	43 (25.7%)	13 (19.4%)	30 (30.0%)	
I was afraid of the unknown, n (%)				
Not at all	46 (27.5%)	17 (25.4%)	29 (29.0%)	0.666a
Somewhat	30 (18.0%)	12 (17.9%)	18 (18.0%)	
Moderately	37 (22.2%)	16 (23.9%)	21 (21.0%)	
Very much	54 (32.3%)	22 (32.8%)	32 (32.0%)	
I was worried about my baby's health, n (%)				
Not at all	38 (22.8%)	18 (26.9%)	20 (20.0%)	0.431a
Somewhat	17 (10.2%)	5 (7.5%)	12 (12.0%)	
Moderately	52 (31.1%)	22 (32.8%)	30 (30.0%)	
Very much	60 (35.9%)	22 (32.8%)	38 (38.0%)	
I was worried about making medical decisions, n (%)				
Not at all	46 (27.5%)	19 (28.4%)	27 (27.0%)	0.498a
Somewhat	30 (18.0%)	16 (23.9%)	14 (14.0%)	
Moderately	49 (29.3%)	15 (22.4%)	34 (34.0%)	
Very much	42 (25.1%)	17 (25.4%)	25 (25.0%)	
I was worried about my ability to tolerate the pain, n (%)				
Not at all	39 (23.4%)	14 (20.9%)	25 (25.0%)	0.746a
Somewhat	29 (17.4%)	12 (17.9%)	17 (17.0%)	
Moderately	55 (32.9%)	24 (35.8%)	31 (31.0%)	
Very much	44 (26.3%)	17 (25.4%)	27 (27.0%)	

After adjustment for maternal age, parity, and the presence of chronic disease, having a birth companion was not significantly associated with prolonged labor (>12 hours), cesarean delivery, need for neonatal medical care, skin-to-skin contact, or delayed initiation of breastfeeding beyond one hour (all p-values >0.05). However, the presence of a birth companion was independently associated with higher odds of episiotomy (adjusted OR: 5.40, 95% CI: 2.06-14.87; p=0.003). Among the covariates, higher parity was consistently associated with lower odds of prolonged labor and episiotomy, while maternal chronic disease was associated with increased odds of prolonged labor. Older maternal age was independently associated with increased odds of cesarean delivery and reduced likelihood of skin-to-skin contact. No other adjusted associations reached statistical significance after correction for multiple testing (Table 6).

**Table 6:** Multivariable binary logistic regression for categorical maternal and fetal outcomes Abbreviations: CI = Confidence interval, OR = Odds ratio a: *p<0.05; b: Benjamini & Hochberg correction for multiple testing

Characteristic	Adjusted OR (95% CI)	p-value a	q-value b,a
Prolonged labor >12 hours			
Had a companion during delivery	1.10 (0.43-2.67)	0.841	0.841
Mother's age (Years)	1.01 (0.94-1.08)	0.779	0.841
Parity	0.32 (0.11-0.85)	0.024*	0.049*
Having a chronic disease	3.78 (1.43-10.16)	0.007*	0.029*
Cesarean delivery			
Had a companion during delivery	0.43 (0.09-1.59)	0.236	0.253
Mother's age (Years)	1.11 (1.02-1.22)	0.018*	0.072
Parity	0.40 (0.09-1.88)	0.226	0.253
Having a chronic disease	2.03 (0.55-6.56)	0.253	0.253
Episiotomy			
Had a companion during delivery	5.40 (2.06-14.87)	0.001*	0.003*
Mother's age (Years)	0.99 (0.91-1.08)	0.782	0.782
Parity	0.08 (0.02-0.24)	<0.001*	<0.001*
Having a chronic disease	2.59 (0.66-9.95)	0.165	0.288
Prolonged labor >12 hours	1.79 (0.65-4.87)	0.255	0.357
Weight of baby<2500 g	0.29 (0.06-1.31)	0.12	0.28
Need for neonatal medical care	0.87 (0.35-2.18)	0.771	0.782
Need for neonatal medical care			
Had a companion during delivery	1.28 (0.61-2.71)	0.515	0.572
Mother's age (Years)	1.03 (0.97-1.09)	0.417	0.572
Parity	0.77 (0.31-1.91)	0.572	0.572
Having a chronic disease	2.11 (0.78-5.74)	0.143	0.57
Gestational age (Full term)	0.14 (0.01-3.43)	0.228	0.57
Skin-to-skin contact			
Had a companion during delivery	0.96 (0.45-2.06)	0.921	0.921
Mother's age (Years)	0.92 (0.86-0.98)	0.008*	0.034*
Parity	2.19 (0.89-5.62)	0.095	0.189
Having a chronic disease	0.51 (0.18-1.34)	0.184	0.245
Delayed breastfeeding beyond 1 hour			
Had a companion during delivery	1.82 (0.86-3.94)	0.118	0.277
Mother's age (Years)	1.05 (0.99-1.11)	0.138	0.277
Parity	0.89 (0.36-2.16)	0.791	0.95
Having a chronic disease	0.97 (0.38-2.46)	0.95	0.95

As regards the total modified STAI score, the multivariable linear regression model showed that having a birth companion was independently associated with a significantly higher total score, with an adjusted increase of 5.16 points compared to women without a birth companion (95% CI: 3.32-7.00, p<0.001). Among the other covariates, older maternal age was associated with a modest increase in the total score, whereas higher parity was associated with a lower total score. The presence of a chronic disease was not significantly associated with the total score after adjustment (Table 7).

**Table 7:** Multivariable linear regression for the total modified State-Trait Anxiety Inventory (STAI) score Abbreviation: CI = Confidence interval a: *p<0.05; b: Benjamini & Hochberg correction for multiple testing

Characteristic	Adjusted Beta (95% CI)	p-value a	q-value b,a
Total score			
Had a companion during delivery	5.16 (3.32-7.00)	<0.001*	<0.001*
Mother's age (Years)	0.15 (0.01-0.30)	0.037*	0.050*
Parity	-2.43 (-4.61 to -0.25)	0.029*	0.050*
Having a chronic disease	0.68 (-1.60 to 2.96)	0.557	0.557

## Discussion

The presence of a birth companion is a right of every pregnant woman during childbirth [2,3]. However, several institutions lack a clear policy regarding the implementation of this right practice [15], despite the reported benefits to the mother, newborn, and health staff [11,13,14].

We found that, during the study period, 67 women participated in the Safe Childbirth Initiative, and so they had a birth companion, accounting for 40.1% of women giving birth during this time period. A previous study in Riyadh, Saudi Arabia, in 2013 reported that 14.2% of Saudi women had ever had a supportive companion during labor, while 45.3% preferred the presence of a birth companion [17]. A more recent study in Al-Ahsa, Saudi Arabia, reported that 43.2% of women giving birth had a companion during delivery, which agrees with our results [18]. This shows an increasing awareness of the importance of childbirth companions owing to the efforts exerted by health authorities under the Safe Childbirth Initiative [16,21], which resulted in a much higher implementation of this practice in the current study.

Meanwhile, several higher rates of the presence of birth companions have been reported by studies in other countries. One study in the United Arab Emirates stated that 77% of women undergoing childbirth preferred the presence of support during labor [26]. Another study in Oman reported that 86.26% of women had a birth companion [27]. The implementation of birth companionship is generally sub-optimal in several regions, low- and middle-income countries [28]. According to the results of a recent scoping review, coverage was below 40% in one-third of included studies and between 40% and 80% in another third [28]. Implementation of birth companionship is facilitated by the availability of legal frameworks that allow companions either throughout labor or only at specific stages of birth [15,29,30].

We explored the causes for the lack of a birth companion among our sample, and the most reported cause was the hospital not offering a choice (44, 44%) of women with no birth companion. Other causes included the feeling of multiparous women that they could handle it alone (n=24, 24%), feeling more privacy (n=9, 9%), the lack of available suitable companions (n=7, 7%), and fear of embarrassment (n=5, 5%). In 2% (n=2) of cases, the husband's refusal was the cause. These causes accord with those previously reported in other studies. In Saudi Arabia, most public hospitals have no clear policies regarding the presence of a childbirth companion. Moreover, even if the hospital policy allows, the implementation of birth companions faces challenges owing to the absence of a suitable family member, and the hospital is not able to provide one due to the shortage of nursing staff. This leads to leaving pregnant women alone for variable intermittent periods during the early phases of labor [17]. In addition, the other reported causes, such as the fear of embarrassment, lack of privacy, and the husband’s refusal, likely arise from the conservative nature of Saudi society and religious teachings, which limit the exposure of the human body to necessary circumstances only.

The birth companion can be a family member, partner, friend, midwife, or nurse. In the present study, the companion was the woman’s mother or sister in 68.7% (n=46) of cases and the husband in 19.4% (n=13). This finding agrees with the results of previous studies. One study on Tanzanian women stated that mothers were the most common birth companions, accounting for 34.5% of cases [31]. In addition, studies conducted in Saudi Arabia and Oman found that mothers were the birth companions of choice by 58%, 59%, and 30.86% of participants, respectively [17,27]. This can be explained by the intimacy between daughters and mothers in general, so the women undergoing delivery feel more comfort and safety in the presence of their mothers [32]. In addition, women’s mothers have experienced the pains and stresses of labor, so they understand the needs of their daughters during childbirth and are thus usually able to provide the required emotional and physical support during and after childbirth. Moreover, social norms in most Arab communities favor the presence of mothers with their daughters during childbirth.

In the present study, women with a birth companion were younger, more educated, and more often primiparous. This could be partially explained by the higher awareness of highly educated women of their right to have a companion. In addition, primiparous women might seek additional support due to the stress and worry felt when they undergo delivery for the first time, and the concerns of the family about the well-being and health of the inexperienced mother and the newborn. These findings highlight the importance of tailoring the awareness campaigns of birth companionship to address less educated groups and inform them about their right to have a birth companion and the potential benefits.

Research has demonstrated that the presence of a birth companion provides several benefits to the mothers [28]. In the present study, emotional support was offered by companions in the majority of cases (n=66, 98.5%). Additionally, companions encouraged exercise and breathing and performed a massage that helps to alleviate pain and stress in 35.8% (n=24) and 32.8% (n=22) of cases, respectively. Similar forms of support were reported by previous studies [5,33-35].

In our study, there was no significant difference between women with and without birth companions as regards the delivery method or duration. This may be explained by the standardized obstetric management at our institution, so the decision regarding labor management and the choice of cesarean section, which are made based on evidence-based medical indications, are less likely to be influenced by the presence of a birth companion.

Episiotomy was done in a significantly higher percentage of women with a companion (adjusted OR=5.40, 95% CI: 2.06-14.87, p=0.003), which may be attributed to other unmeasured factors such as fetal positioning, fetal distress, prolonged second stage of labor, the use of regional analgesia, and institutional protocols [36]. Meanwhile, there is a potential impact of provider-driven practices, as clinicians might perform episiotomy more readily when a companion is present, either to expedite delivery or in response to maternal expectations. Additionally, companions may influence maternal positioning or pushing techniques.

We found no significant difference between the two studied groups regarding neonatal outcomes, including birth weight, the need for specialized care, the establishment of skin-to-skin contact, or the time of initiating breastfeeding. These findings are in contrast to previous research, which reported that birth companionship enhances the physiological process of labor, leading to shorter duration of labor, higher rate of unassisted vaginal delivery, and satisfactory five-minute Apgar scores [6,10]. The lack of significant difference regarding these outcomes in the present study may be partially attributed to the relatively small and unbalanced sample size between the two groups. In addition, some outcomes, such as skin-to-skin contact and the time of initiating breastfeeding, may be susceptible to cultural concepts or hospital routines. A national cross-sectional study from Saudi Arabia reported low rates of 43.4% for the early initiation of breastfeeding, and that the rates differed significantly according to health sectors, being lower in Ministry of Health facilities [37]. These results warrant proper training of health staff and raising the awareness of pregnant women and their expected birth companions regarding the importance of initiating skin-to-skin contact with the newborn and timely breastfeeding.

In the current study, participants with birth companions had a significant tendency to feel calmer, emotionally supported, and safer, and experienced less anxiety (p<0.05). These results agree with those of previously conducted studies worldwide [8,27,38,39]. A systematic review that was based on 51 studies found that women with birth companions felt safe, strong, confident, and secure [5]. Mothers who had no birth companions reported feeling lonely and isolated [30], as well as suffering depression [40]. Stress during childbirth stimulates the sympathetic nervous system and decreases the secretion of endogenous oxytocin, resulting in weakening or cessation of uterine contractions. Therefore, ensuring a calm and safe environment as well as the alleviation of anxiety during labor can enhance the release of endogenous oxytocin [41]. Fear of childbirth has been associated with increased rates of elective cesarean sections [42,43].

The present study showed several points of strength, including the exploration of the causes underlying the absence of birth companions among a sample of Saudi women. Additionally, the present study assessed the potential association between birth companionship and a variety of outcomes related to both the mother and newborn. Meanwhile, our study was subject to some limitations. A potential risk of recall bias exists arising from the study design as a retrospective observational study in which some outcomes were self-reported. The timing of interviews varied among participants after delivery; the risk of recall bias may be higher among those interviewed several months after delivery compared to those interviewed one month postpartum. Although the investigators attempted to minimize the risk of recall bias by using a standardized data collection sheet, training of interviewers, and checking self-reported outcomes with hospital records, the possibility of recall bias cannot entirely be eliminated. In addition, the study was conducted on women giving birth in a single center in Al-Ahsa, so the results may not be generalizable to other geographic regions. While subgroup analyses may have provided additional insights, small sample sizes within strata precluded reliable statistical inference and thus were not undertaken. Moreover, the results obtained from observational studies - such as the present study - are associations that cannot imply causality, which requires a different study design (e.g., clinical trials) and dictates the presence of crucial criteria defined by Hill, including temporality, consistency, and specificity [44].

## Conclusions

The presence of a birth companion is associated with less anxiety, calmness, and feeling emotionally supported during delivery. The assessment of the potential impact of birth companionship on other maternal and fetal outcomes requires adjustment for the effects of other confounding factors, such as fetal distress, fetal position, and institutional protocols. Preferably, future studies should consider propensity score matching for balancing the distributions of confounders between the studied cohorts. Prospective cohort or quasi-experimental designs should be considered in future research. Meanwhile, birth companionship should be highlighted as a right for every woman and should be incorporated in the policies of all health facilities. Educational initiatives directed at healthcare providers, pregnant women, and prospective birth companions can improve understanding of birth companionship and its potential roles during labor and delivery, and increase the acceptability of birth companionship.
